# Childhood trauma and subclinical PTSD symptoms predict adverse effects and worse outcomes across two mindfulness-based programs for active depression

**DOI:** 10.1371/journal.pone.0318499

**Published:** 2025-01-30

**Authors:** Nicholas K. Canby, Elizabeth A. Cosby, Roman Palitsky, Deanna M. Kaplan, Josie Lee, Golnoosh Mahdavi, Adrian A. Lopez, Roberta E. Goldman, Kristina Eichel, Jared R. Lindahl, Willoughby B. Britton

**Affiliations:** 1 Department of Psychiatry and Human Behavior, Warren Alpert Medical School, Brown University, Providence, RI, United States of America; 2 Department of Religious Studies, Brown University, Providence, RI, United States of America; University of Cologne: Universitat zu Koln, GERMANY

## Abstract

Within mindfulness-based programs (MBPs), mixed results have been found for the role of childhood trauma as a moderator of depression outcomes. Furthermore, childhood trauma and PTSD symptoms have been identified as possible risk factors for the occurrence of meditation-related adverse effects (MRAE). The present research examined multiple forms of childhood trauma and PTSD symptoms as predictors of depression treatment outcomes and MRAEs. Various forms of childhood trauma (e.g., abuse and neglect) were examined as predictors of depression treatment outcomes and participant attrition using secondary analyses of two MBP clinical trials (*N* = 52 and 104, respectively). Study 2 also examined meditation-related side effects (MRSE) and MRAE as outcomes and current subclinical and past PTSD symptoms as predictors. Childhood trauma led to worse depression outcomes across both study 1 and study 2, such that total childhood trauma and childhood sexual abuse were significant predictors across both studies. Childhood sexual abuse predicted attrition in study 2. Finally, multiple forms of childhood trauma and PTSD symptoms predicted MRSE, while total childhood trauma, childhood emotional abuse, and subclinical PTSD symptoms predicted lasting MRAE. Childhood trauma and PTSD symptoms may lead to worse outcomes and a greater occurrence of adverse effects within MBPs for active depression. These results call for further trauma-sensitive modifications, safety monitoring, participant screening, and provider education when implementing these programs.

## Introduction

Mindfulness Based Cognitive Therapy (MBCT) is an evidence-based treatment for the prevention of recurrent depression that combines cognitive behavioral therapy with mindfulness meditation practice [1]. Designed to prevent relapse of major depression [2], MBCT was developed particularly for individuals with a history of multiple prior depressive episodes in remission from active depression at the time of treatment [3]. Increasingly, MBCT has been extended beyond its originally intended purpose of prophylaxis to be offered to populations with *active* affective disturbances [4]. As MBCT reaches wider populations, recent scholarship has begun to identify individual-difference determinants of differential response to treatment and risk factors for meditation-related adverse effects [MRAEs; 5, 6]. Research has identified childhood trauma history as a potential treatment moderator of interest [7], as well as a possible risk factor for the occurrence of MRAEs [8].

### Childhood trauma as a moderator of MBCT-related depression outcomes

Childhood trauma has been examined as a moderator of MBCT’s efficacy on depression relapse prevention, primarily among individuals in remission from major depression [9–12]. Ma and Teasdale [11] found that MBCT was more effective at reducing depression relapse among individuals with higher levels of childhood trauma. In the first dismantling study of MBCT, Williams, Crane [12] reported an overall finding of no difference between MBCT, a cognitive-psychological intervention, or continuing treatment as usual. However, a subgroup analysis showed that individuals with a history of childhood trauma were less likely to relapse if they received MBCT. Similarly, Kuyken, Hayes [10] also found that a modified MBCT with antidepressant taper outperformed maintenance anti-depressants in preventing relapse, but only for a subgroup of individuals with high childhood trauma. These findings suggest benefits in preventing relapse among individuals with histories of childhood trauma and low levels of current depression.

The response of individuals with a history of childhood trauma to MBCT for *active* depression is less well understood. Qualitative data from a study using MBCT as a treatment for active depression found that many individuals who had residual depression symptoms post-treatment had a history of childhood trauma, specifically sexual abuse or emotional neglect [13]. Moreover, an RCT investigating MBCT for active treatment-resistant depression showed reduced likelihood of treatment response and remission in individuals with a history of childhood emotional abuse [14]. These studies suggest that mindfulness-based programs (MBPs) may present difficulties for individuals with active depression and a history of childhood trauma. While several studies have demonstrated significant reduction in depression symptoms after an MBP in samples with PTSD [15] or childhood trauma [16], few studies have examined childhood trauma specifically as a moderator of active depression outcomes.

### Childhood trauma as a predictor of meditation-related adverse effects (MRAEs)

In addition to moderating depression outcomes, childhood trauma may also relate to the occurrence of MRAEs. Though widely underreported and insufficiently monitored in meditation research [17, 18], MRAEs with varying degrees of duration and impairment have been documented in the context of MBPs [18–22] and in non-clinical settings [23–26]. Because frequencies of MRAEs depend on how adverse effects are defined and measured, frequency estimates range widely across studies [18, 19]. The most valid and conservative estimates, based on an epidemiological study of US adults, found that half of individuals who had tried some form of meditation at least once reported at least one MRAE and that one in ten reported an MRAE that lasted more than one month or was associated with impairment in functioning [8]. Within an MBP, Britton, Lindahl [18] found that the number of participants who experienced MRAEs varied widely depending on the criteria used to define them, which included valence, impact, and duration. While 83% of participants in Britton, Lindahl [18] experienced at least one meditation-related side effect (MRSE), defined as any unintended experience during or following meditation that was not the goal of treatment, experiences involving negative valence, negative impact, or lasting bad effects occurred in 58%, 37%, and 6–14% of the sample, respectively.

While the existence of MRAEs is now firmly established, research on individual difference risk factors for MRAEs is in its early stages. Theoretically, individuals with a history of trauma may be more prone to experiencing MRAEs since documented symptoms involve dysregulated arousal (a characteristic of PTSD), dissociative states, and the re-experiencing of traumatic memories [18, 27]. While the empirical literature linking trauma history to MRAEs is sparse, emerging evidence suggests an association. For example, Goldberg, Lam [8] found that respondents with a greater history of childhood trauma were more likely to have experienced at least one MRAE, a greater number of MRAE, and impairment due to meditation [8]. Additionally, Zhu, Wekerle [28] conducted a study of college students that evaluated the correlations between participants’ history of childhood trauma and trauma-related symptoms with their affective response to a single 5-minute focused attention mindfulness practice. This study found that childhood trauma predicted participants’ difficulty attending to their breath. They also found that participants’ current trauma-related symptoms were associated with increased post-meditation distress and traumatic re-experiencing. While both of these studies suggest a relationship between trauma and MRAE within non-clinical samples, no study to date has examined whether, and what kind of, trauma impacts participants’ risk for MRAE in the context of an MBP for active depression.

The present research sought to assess the impact of childhood trauma and past/current PTSD symptoms on MBP treatment outcomes and MRAEs in participants with active depression across two separate clinical trials. Study 1 investigated the role of childhood trauma as a predictor of pre-post changes in active depression symptoms in a randomized controlled trial of MBCT. We hypothesized that a more severe history of childhood trauma would be associated with worse depression outcomes and greater participant dropout. Study 2 investigated this same hypothesis in the context of a three-armed dismantling study of MBCT for active depression with a larger sample and more established measure of childhood trauma. Study 2 also investigated whether childhood trauma predicted increased MRSE, negative valence MRAE, negative impact MRAE, and lasting bad effects (LBEs), as defined by Britton, Lindahl [18]. As such, we hypothesized that more severe levels of childhood trauma and current/past PTSD symptoms would be associated with a greater occurrence of meditation-related side effects and adverse effects.

## STUDY 1: A pilot study of the effects of childhood trauma on MBCT outcomes

### Methods

#### Participants

Individuals with at least some degree of active symptoms of recurrent unipolar depression were recruited for a study examining the effects of MBCT vs. waitlist control condition. Inclusion criteria were: age 18+, meeting DSM-IV criteria for major depression in the last 60 months, a lifetime history of at least three major depression episodes, and being in partial remission during the past 8 weeks. Partial remission was defined by a subjectively reported improvement in symptoms in the past two months and a lack of severely depressed mood, severe anhedonia, or suicidal ideation [for details see 29]. Individuals on antidepressant medication (ADM) were permitted to participate if there was no change in ADM during the three months prior to enrollment or the active phase of the study.

#### Procedure

Study recruitment took place via community advertisements between January 1^st^, 2004 and June 30^th^, 2005. After providing written informed consent, eligible participants completed a packet of self-report questionnaires including demographic information, a personal history form, and the Beck Depression Inventory [BDI; 30]. Participants then completed a laboratory-based assessment including the clinician-rated Structured Clinical Interview for the Diagnostic and Statistical Manual of Mental Disorders-IV [SCID-IV; 31]. After completing these baseline assessments, participants were then randomized to either an 8-week MBCT program or a wait list control condition, which took place between July 2004 and December 2005. Participants randomized to receive MBCT attended weekly 3-hour sessions and an all-day retreat, as well as homework for individual practice [29]. After the MBCT program, participants again completed the BDI. The University of Arizona Institutional Review Board approved the study protocol (BSC#03–63).

#### Measures

The Beck Depression Inventory (BDI) is a 21-item self-report measure of depression symptoms (*α* = .81 pre-treatment, .90 post-treatment).

A short personal history form adapted from related measures [32, 33] queried potentially traumatic events by asking participants to fill out a series of checklists about whether (and if so when) they had ever experienced the following: “Life-threatening illness or injury,” “physical/sexual abuse,” “Assault or rape,” and “Loss of a loved one (relation ________).” This question was followed by an item querying: “Most traumatic event” with several lines provided for an open response.

The SCID-IV [31] is a structured clinical interview for establishing DSM-IV diagnoses. As part of the PTSD module, the interviewer systematically queried past history of potentially traumatic events, with follow-up questions focused on establishing the nature, time, and impact of the events. These were recorded as interview notes, which were used as material for the current analysis.

*Calculation of CTQ-BAS*. A directed qualitative analytic approach tailored for this study was used to extract an index of childhood trauma from the SCID-IV interview and the personal history form. To align with prior research that used the Childhood Trauma Questionnaire [CTQ; 34] as a predictor of response to MBCT [12], codes corresponding with the types of childhood trauma measured by this scale were generated. These codes were summed for a total Childhood Trauma Questionnaire-Based Adversity Score (CTQ-BAS) and four CTQ-BAS subscales (physical abuse, emotional abuse, sexual abuse, and physical neglect). See S1 File for more information about the coding process and S1 Table for the list of codes.

#### Analysis

Analyses were conducted in SPSS Version 28 with bootstrapping (5,000 iterations), following recommendations by [35] to improve estimates of the sample distribution. Missing data due to participant attrition were excluded from analyses when predicting ΔBDI, while prediction of participant attrition included the entire sample. There was no missing data in baseline measures. Overall differences in ΔBDI between MBCT and waitlist were tested via an independent samples *t* test. Associations between CTQ-BAS variables and pre-post trial ΔBDI were tested in multiple linear regression while controlling for treatment condition (MBCT or waitlist) to account for treatment effects. To illustrate effects in each condition, follow-up analyses tested associations of CTQ-BAS and ΔBDI within the MBCT and waitlist conditions separately. The primary regression analyses were powered at .81 to detect an effect of .4 (small-medium effect). Participant attrition was predicted using Firth’s penalized likelihood logistic regression (as further described in study 2) with CTQ-BAS variables as predictors.

### Results

A total of 52 participants were randomized to the MBCT program (*N* = 29) or waitlist control (*N* = 23). Seven participants dropped out before study completion (3 from MBCT, 4 from waitlist control). The total sample had the following characteristics: *M* age = 47.36 (*SD* = 9.09), *M* years of education = 16.90 (*SD* = 2.05), sex = 79% female, *M* baseline BDI = 9.41 (*SD* = 5.89). See Table 1 for descriptive statistics for CTQ-BAS variables, none of which differed at baseline between the treatment and control conditions (*p* < .05). No differences were found between treatment groups or completers vs. non-completers on age, sex, use of antidepressant medication, depression severity, or previous months of depression (*p* < .05). See Britton, Shahar [29] for more information. An independent-samples *t*-test revealed that the MBCT group reported greater reduction in BDI (M_ΔBDI_ = -4.48) than the control group (M_ΔBDI_ = 1.53), *t*(43) = -3.05, *p* = .004, 95% CI = -2.03, -9.98. On average, BDI scores were reduced by 6.01 more points in the MBCT group than the waitlist (SD = 1.97).

**Table 1:** Study 1: Descriptive statistics for childhood trauma.

Trauma at baseline		*M (SD)*	% Sample > 0
	CTQ-BAS total	1.13 (*1*.*61*)	44.2
	CTQ-BAS physical abuse	0.12 (*0*.*32*)	11.5
	CTQ-BAS emotional abuse	0.19 (*0*.*40*)	19.2
	CTQ-BAS sexual abuse	0.71 *(1*.*24*)	26.9
	CTQ-BAS physical neglect	0.12 (*0*.*32*)	11.5

*Note*. *N* = 52. CTQ-BAS = Childhood Trauma Questionnaire-Based Adversity Score, which was calculated with information obtained through the SCID-IV interview and personal history form. Please note that the CTQ-BAS does not include some of the items from the original CTQ (e.g., reverse scored items, see S1 Table for more information). As a result, the emotional abuse and physical neglect subscales were created by summing 4 items instead of 5, and the emotional neglect and denial subscales were not possible to calculate.

Table 2 displays the results of multiple linear regression models predicting ΔBDI from CTQ-BAS variables while adjusting for treatment condition. CTQ-BAS total, physical abuse, and sexual abuse were associated with worse ΔBDI overall across both treatment conditions. On average and while controlling for the effects of condition, the endorsement of a single CTQ-BAS category was associated with an increase in pre-post BDI score of 2.47 points for CTQ-BAS total, 7.75 points for CTQ-BAS physical abuse, and 2.73 points for CTQ-BAS sexual abuse. The interaction effect between CTQ-BAS total and treatment condition was non-significant (*b* = 0.79, 95% CI: -1.49, 3.072, *p* = .488), while subscale interaction effects were not possible to calculate due to sample limitations. Follow-up analyses examined CTQ-BAS variables as predictors of ΔBDI within each condition separately. Results indicated that CTQ-BAS total, physical abuse, emotional abuse, and sexual abuse were associated with less improvement or worsening of depression scores in the MBCT condition, while only CTQ-BAS total and CTQ-BAS sexual abuse were associated with less improvement or worsening of depression scores in the control group (see S2 Table for details). None of the CTQ-BAS variables significantly predicted participant attrition (see S3 Table for more information).

**Table 2:** Study 1: Trauma variables as predictors of pre-to-post BDI changes.

		Prediction of ΔBDI			
Predictors:		*R* ^2^	*b*	95% CI	
				*LL*	*UL*
Model 1:		.44***			
	CTQ-BAS Total		**2.47** ***	**1.40**	**3.49**
	MBCT vs. Control		-6.10***	-9.27	-2.95
Model 2:		.52**			
	CTQ-BAS Physical Abuse		**7.75** *	**-0.88**	**16.68**
	MBCT vs. Control		-6.49**	-10.49	-3.06
Model 3:		.46**			
	CTQ-BAS Emotional Abuse		4.70	-0.52	10.52
	MBCT vs. Control		-5.56**	-9.52	-1.77
Model 4:		.40***			
	CTQ-BAS Sexual Abuse		**2.73** ***	**1.36**	**4.03**
	MBCT vs. Control		-6.53***	-9.61	-3.31
Model 5:		.43*			
	CTQ-BAS Physical Neglect		1.69	-5.70	7.90
	MBCT vs. Control		-5.81**	-9.63	-2.16

Note: *N* = 45 and *df* = 2, 42 for all models. Stars next to *R*^2^ refer to significance of *F* statistic for total model fit. CTQ-BAS = Childhood Trauma Questionnaire-Based Adversity Score; CI = confidence interval; LL = lower limit; UL = upper limit; Coefficients were calculated through bootstrapping with 5,000 iterations.

*p < .05

**p < .01

*** p < .001.

## STUDY 2: Replication of pilot results and investigation of adverse effects

### Methods

#### Participants

Participants were English-speaking, between the ages of 18–65, and exhibited mild to severe levels of depression [score of 10–48 on The Inventory of Depressive Symptomatology—Clinician Rated; IDS-C] [36] or high level of negative affect [negative affect > 18 in last 30 days on Positive and Negative Affect Schedule–Expanded Form; PANAS-X] [37]. While participants with a current post-traumatic stress disorder (PTSD) diagnosis were excluded from the study, those with a past episode of PTSD or current subclinical levels of PTSD were allowed to participate. See Britton, Davis [38] for details.

#### Procedure

This study was part of a larger dismantling study in which participants were randomly assigned to either a standard MBCT intervention or two single component variants: a focused attention (FA) intervention, or an open monitoring (OM) intervention. The MBCT module followed the standard session-by-session manual [39], while the FA and OM curriculums emphasized specific forms of meditation that are both present in standard MBCT. All three interventions were matched for duration and format (e.g., class size, number of handouts, minutes of meditation). Classes met for three hours once a week for 8 weeks with a daylong retreat during the 7th week. Homework consisted of 45 min per day of formal meditation practice. See [38] for more information.

Following an initial telephone screening, all participants completed an in-person assessment including the SCID-IV, the IDS-C, and the PANAS-X to confirm their eligibility. Eligible participants were randomized to one of the three treatment types and participated in one of nine treatment groups, three groups for each treatment type. Online questionnaires were administered at baseline (week 0), week 4, post-course (week 8) and three-month-follow-up (week 20). These questionnaires included the CTQ [baseline only; 34] and the Quick Inventory of Depressive Symptomatology [QIDS; all four time points; 40]. Participants returned to the lab for clinician administered assessments at post-course (week 8) and three-month-follow-up, which included the IDS-C at both timepoints and the Meditation Experiences Interview [MedEx-I; 18] at three-month-follow-up only.

This registered clinical trial (clinicaltrials.gov #NCT01831362) was conducted at Brown University in Providence, Rhode Island, between November 1^st^, 2012 and March 31^st^, 2016. It was approved and supervised by the Brown University Institutional Review Board (#1105000399), an independent Data Safety Monitoring Board and the National Center for Complementary and Integrative Health’s Office of Clinical and Regulatory Affairs. Eligible participants provided written, informed consent and did not receive financial compensation.

#### Measures

*Trauma history and symptoms*. The CTQ is a validated 28-item self-report inventory measuring experiences of abuse and neglect during childhood and adolescence [34]. The questionnaire is composed of five 5-item subscales: Physical Abuse (*α* = .79), Emotional Abuse (*α* = .86), Sexual Abuse (*α* = .95), Physical Neglect (*α* = .71), and Emotional Neglect (*α* = .93), which combine to form a total score (*α* = .85) that reflects overall abuse and neglect history. Participants respond to a series of statements about childhood experiences using options that range from 0 (Never True) to 4 (Very Often True) on a Likert-type scale. The CTQ also contains a 3-item Minimization/Denial subscale (*α* = .29), intended to detect underreporting of trauma in order to provide socially desirable responses. While a simple cutoff score has not been established for this subscale, prior research recommends the exclusion of participants from analysis who have high minimization/denial scores [41].

The SCID-IV [31] was used in this study to determine whether participants fit criteria for current symptoms of subclinical PTSD and/or a past diagnosis of PTSD. Dichotomous variables were calculated to account for these diagnostic presentations.

*Outcome measures*. The QIDS is a 16-item (*α* = .72) self-report questionnaire designed to measure the presence and severity of the nine characteristic symptoms of a major depressive episode as defined by the *DSM-IV* [40]. Symptoms are rated on a scale of 0–3 (not present to severe or constantly present) in terms of the past seven days. Total scores range from 0–27.

The IDS-C is a 30-item clinician-administered interview that also measures symptoms of depression in the past seven days, as defined by the *DSM-IV*. It is highly similar to the QIDS, yet differs in that it contains more items and is administered by a clinician. Scores range from 0–90, such that 0–13 is considered normal, 14–25 mild, 26–38 moderate, 39–48 severe, and 49–84 very severe. The IDS-C was administered by graduate-level research assistants who were trained by PhD-level clinicians and met high inter-rater reliability (κs at week 0, week 8, and week 20 = 0.89, 0.93, 0.94, respectively). Both the IDS and QIDS were included in the present study due to research indicating that self-reported and clinician administered depression measures often produce differing effect sizes in psychotherapy outcomes research [42].

The MedEx-I [18] is a structured interview designed to assess the presence of MRSE/MRAE. It begins with the following open-ended query: “Have you had any unusual, unexpected, unpleasant or challenging experiences or side-effects of meditation?” This is followed by 44 probes that assess specific experiences since the beginning of the program within the following six clusters: cognitive, perceptual, sense of self, affective, somatic/physiological, and social/occupational. Each time such an experience is endorsed, follow-up questions are posed about its duration, valence, impact, and causal connection to meditation practice. Mediation-related side effects (MRSE) refer to any unintended effects of meditation, regardless of valence. Mediation-related adverse effects (MRAE) refer to all MRSE with a negative valence and are assessed in 3 tiers: 1) A negative valence MRAE is experienced as unpleasant while it is occurring, regardless of its impact on functioning; 2) A negative impact MRAE results in a negative impact in functioning, requires countermeasures or a change in behavior, and 3) Lasting bad effects (LBEs) were defined as negative impact MRAEs with three possible durations: more than one day, more than one week, and more than one month. Scoring involved summing the number of experiences for MRSEs, negative valence MRAEs, and negative impact MRAEs. Dichotomous variables were used to represent whether participants experienced LBEs lasting more than a day, week, or month. Finally, participant descriptions were recorded, transcribed, and queried for quotes relating to trauma symptoms. Illustrative quotes were selected and included in the results as examples.

#### Analysis

Data were analyzed using SPSS 28 and R 4.2.2. The handling of missing data and outliers is described in S2 File. Different statistical models were used for each of the three types of outcome variables investigated: longitudinal continuous variables (QIDS/IDS) were examined with multilevel growth curve models; count variables (MRSE/MRAE) were examined with multilevel generalized linear models, and binary variables (attrition and LBEs) were examined with Firth’s penalized likelihood logistic regression models. As based on recommendations from [43], multilevel modeling was used to account for the nested structure of the data for the first two model-types, such that multiple timepoints were nested within each individual participant (in models involving longitudinal responses), individual participants were nested within the nine treatment groups (for all models), and groups were nested within the three treatment types (for all models). Random intercepts that accounted for no variance (ICC < .001) were not retained within models. All trauma variables (CTQ total and subscales, current subclinical PTSD, history of PTSD) were examined independently as fixed predictors of each of these outcomes. All analyses were run both with the full sample and with a subset of the data that excluded individuals who scored above 3.0 and 3.5 on the CTQ denial subscale, as recommended by MacDonald, Thomas [41]. See S3 File for more information about these statistical models.

### Results

#### Sample characteristics and preparatory analyses

A total of 104 participants were cluster randomized into nine groups, three for each of the three treatments (MBCT, FA, and OM). Eight participants dropped out of the study after randomization (see S2 File for more detail). The total sample had the following characteristics: *M* age = 40.24 (*SD* = 12.82), *M* years of education = 17.20 (*SD* = 2.68), sex = 74% female. As displayed in Table 3, participants had mild to severe levels of depression symptoms, with a wide range of previous episodes, and a third of the sample on antidepressant medication. While only 15% of the sample met criteria for current subclinical PTSD, 95% of the sample endorsed at least one category of childhood trauma. Emotional neglect and emotional abuse were the most commonly endorsed forms of childhood trauma (90% and 85% of the sample, respectively), while sexual abuse was the least commonly endorsed form of childhood trauma (23% of the sample).

**Table 3:** Study 2: Descriptive statistics for depression, trauma, and meditation-related side effects.

Depression at baseline		*M (SD)*	% Sample
	Months depressed	44.01 (*43*.*02*)	
	On antidepressants	-	33.98
	QIDS depression	16.94 (3.77)	
	IDS depression	23.22 (7.34)	
Trauma variables at baseline		*M (SD)*	
	Past PTSD	-	21.36
	Past subclinical PTSD	-	7.77
	Current subclinical PTSD	-	14.56
			% Sample > 0
	CTQ total	0.69 (*0*.*59*)	95.15^a^
	CTQ physical abuse	0.35 (*0*.*56*)	46.60^a^
	CTQ emotional abuse	1.09 (*0*.*97*)	85.44^a^
	CTQ sexual abuse	0.26 *(0*.*63*)	23.30^a^
	CTQ physical neglect	0.40 (*0*.*50*)	56.31^a^
	CTQ emotional neglect	1.34 (*1*.*04*)	90.29^a^
	CTQ denial	1.59 (*1*.*13*)	
Meditation-Related Side Effects		Median # (range)	
	MRSE	2 (0–13)	83.16
	Negative Valence MRAE	1 (0–7)	57.89
	Negative Impact MRAE	0 (0–5)	37.50
	LBE > Day	-	14.29
	LBE > Week	-	9.09
	LBE > Month	-	6.49

*Note*. *N* = 103 for all depression and trauma variables except for QIDS (*N* = 102). *N* = 95 for MRSE and Negative Valence MRAE, *N* = 80 for Negative Impact MRAE, and *N* = 77 for LBE variables. QIDS = Quick Inventory of Depressive Symptomatology; IDS = Inventory of Depressive Symptomatology. CTQ = Childhood Trauma Questionnaire; PTSD = Post-Traumatic Stress Disorder, as measured by the SCID. MRSE = Meditation-related side effects. MRAE = Meditation-related adverse effects as defined by negative valence or negative impact. LBE = lasting bad effect, which was categorized by durations of days, weeks, or months. MRSE and MRAE variables refer to the number of events per person, while LBE variables are dichotomous.

Rates of negative valence and negative impact MRAEs were reported by 58% and 37% of the sample, respectively, while LBEs were reported by 6–14% of participants depending on their duration [18]. The most common MRSEs were vivid imagery or thoughts, perceptual hypersensitivity, somatosensory changes, and traumatic reexperiencing. Similarly, the most common negative valence MRAEs were traumatic reexperiencing, anxiety and panic, perceptual hypersensitivity, affective lability, and agitation. Negative impact MRAEs were most commonly anxiety and panic, agitation, executive dysfunction, and social impairment. Finally, the symptoms that were either constitute or predictive of LBEs were executive dysfunction, insomnia, emotional blunting, self-disturbances, anxiety, time-space distortions, traumatic reexperiencing, derealization, social impairment, and visual lights. See Britton, Lindahl [18] for more descriptive information about MRSE and MRAE, as the present study is a secondary analysis of the same dataset.

Preliminary analyses involving model construction, including testing distributional assumptions and fitting models to the nested data structure, are reported in S4 File. Treatment type did not significantly explain any of the variance in depression outcomes and explained ≤ 1% of the variance in MRSE and MRAE variable.

#### Intervention-related changes in depression

Both QIDS and IDS depression scores significantly improved across the time points measured (*ps* < .0001). On average, QIDS depression scores decreased 2.87 points (*SD* = 3.72) from pre to post treatment and 3.03 points (*SD* = 4.30) from baseline to three-month-follow-up. However, despite the overall improvement, 10 participants (11%) had no change and 14 participants (15%) increased in QIDS depression scores pre-to-post treatment. Similarly, at three-month-follow-up, 6 participants (7%) had no change from baseline and 16 participants (17%) increased in QIDS depression scores relative to baseline. A similar pattern of results was found for the IDS clinician-administered depression scale, which decreased on average 11.82 points (*SD* = 7.79) pre-to-post treatment and 12.04 points (*SD* = 8.08) from baseline to three-month-follow-up. Despite this overall improvement, six participants (6% of sample) increased in IDS-C depression scores pre-to-post treatment, while zero did not change. At three-month-follow-up, one participant (1%) did not change from baseline, while five participants (5% of sample) increased in IDS-C depression scores relative to baseline. See S5 File for detailed results about the construction of depression growth curve models, including linear and polynomial effects of time and growth model parameters for each of these models before trauma predictors were added.

#### Primary analyses

Sensitivity analyses indicated that the exclusion of high CTQ denial scores did not change the pattern of results except by reducing statistical power by lowering sample size. Therefore, only results from the complete dataset are presented.

*Predicting intervention-related changes in depression*. First, the main effect of each trauma variable on depression levels across all time points (the model intercept) was calculated in order to control for this in subsequent analyses. All trauma variables significantly predicted greater depression intercepts for both the QIDS and IDS measures of depression *(p* < .05; see S6 File).

Next, interactions between each trauma variable and linear and quadratic depression slope coefficients were added to each model in order to predict changes in depression over time (see S4 Table). Results indicate that CTQ total (*p* < .05), CTQ sexual abuse (*p* < .05), and CTQ emotional neglect (*p* < .05) significantly predicted intervention-related changes in QIDS, but not IDS, depression scores. Participants with greater levels of CTQ total, CTQ sexual abuse, and CTQ emotional neglect improved less or worsened as a result of the intervention, as measured by self-reported QIDS (but not the clinician-administered IDS) depression scores.

*Predicting meditation-related side effects and adverse effects*. First, each trauma variable was added as a predictor of MRSE/MRAE using the negative binomial generalized linear models described in S4 File. As described in Table 4, all trauma variables significantly predicted a greater number of MRSE and all trauma variables except for physical abuse significantly predicted a greater number of negative valence MRAEs. The number of negative impact MRAEs was significantly predicted by CTQ total, CTQ emotional abuse, CTQ sexual abuse, current subclinical PTSD, and past PTSD diagnosis.

**Table 4:** Study 2: Trauma variables as predictors of number of meditation-related side effects and adverse effects.

	*MRSE*			*Negative Valence MRAE*			*Negative Impact MRAE*		
Predictors:	*b*	95% CI		*b*	95% CI		*b*	*95% CI*	
		*LL*	*UL*		*LL*	*UL*		*LL*	*UL*
CTQ Total	**0.48** **	**0.17**	**0.80**	**0.56** **	**0.17**	**0.96**	**0.67** *	**0.03**	**1.30**
CTQ Physical Abuse	**0.40** *	**0.01**	**0.78**	0.32	-0.18	0.82	0.29	-0.49	1.07
CTQ Emotional Abuse	**0.30** **	**0.12**	**0.48**	**0.32** **	**0.08**	**0.56**	**0.41** *	**0.05**	**0.77**
CTQ Sexual Abuse	**0.40** *	**0.03**	**0.76**	**0.56** *	**0.12**	**1.02**	**0.83** *	**0.14**	**1.52**
CTQ Physical Neglect	**0.48** *	**0.09**	**0.86**	**0.57** *	**0.11**	**1.03**	0.59	-0.16	1.34
Emotional Neglect	**0.18** *	**<0.01**	**0.36**	**0.24** *	**<0.01**	**0.48**	0.17	-0.20	0.55
Current Subclinical PTSD	**0.55** *	**0.06**	**1.04**	**0.95** **	**0.37**	**1.52**	**1.17** **	**0.36**	**1.99**
Past PTSD	**0.60** **	**0.16**	**1.04**	**0.99** ***	**0.49**	**1.48**	**0.95** *	**0.10**	**1.80**

Note: *N* = 95 for MRSE and Negative Valence MRAE models, *N* = 80 for Negative Impact MRAE models. MRSE = Meditation-related side effects; MRAE = Meditation-related adverse effects as defined by negative valence or negative impact; CI = confidence interval; LL = lower limit; UL = upper limit. Dependent variables were modeled as negative binomial count distributions.

*p < .05

**p < .01

*** p < .001.

Next, each trauma variable was added as a predictor of LBEs by duration (days, weeks, months) using Firth’s penalized likelihood logistic regression models. As displayed in Table 5, CTQ total significantly increased the odds of LBE > day, subclinical PTSD significantly increased the odds of LBE > day and LBE > week, and CTQ emotional abuse significantly increased the odds LBE > day, LBE > week, and LBE > month.

**Table 5:** Study 2: Trauma variables as predictors of lasting bad effects and attrition.

	LBE > Day			LBE > Week			LBE > Month			Attrition		
Predictors:	*OR*	95% CI		*OR*	95% CI		*OR*	*95% CI*		*OR*	95% CI	
		*LL*	*UL*		*LL*	*UL*		*LL*	*UL*		*LL*	*UL*
CTQ Total	**3.29** *	**1.08**	**10.78**	3.26	0.88	12.71	2.96	0.65	13.26	1.69	0.54	4.98
CTQ Physical Abuse	1.47	0.37	4.77	1.35	0.22	5.31	1.46	0.18	6.65	1.52	0.35	5.11
CTQ Emotional Abuse	**2.88** **	**1.47**	**6.36**	**2.62** *	**1.24**	**6.08**	**2.63** *	**1.14**	**6.58**	1.09	0.50	2.10
CTQ Sexual Abuse	2.40	0.55	9.33	3.50	0.77	15.31	1.40	0.05	7.04	**3.30** *	**1.28**	**8.32**
CTQ Physical Neglect	2.19	0.60	7.48	2.34	0.50	9.64	2.38	0.40	11.73	2.20	0.60	7.39
Emotional Neglect	1.44	0.79	2.64	1.62	0.79	3.38	1.72	0.75	4.02	1.16	0.58	2.21
Current Subclinical PTSD	**4.76** *	**1.13**	**19.31**	**5.72** *	**1.11**	**28.10**	1.98	0.19	12.25	2.35	0.40	10.42
Past PTSD	3.63	0.88	14.03	2.35	0.39	11.25	1.61	0.15	9.77	2.50	0.54	10.27

Note: *N* = 103 for attrition models, *N* = 77 for LBE variable models. LBE = Lasting Bad Effect; CTQ = Childhood Trauma Questionnaire; PTSD = Post-traumatic Stress Disorder; CI = Confidence Interval; LL = Lower Limit; UL = Upper Limit; OR = Odds Ratio. All models used Firth’s Penalized Likelihood Logistic Regression with dichotomous dependent variables.

*p < .05

**p < .01

*** p < .001.

*Predicting attrition*. Trauma variables were also examined as a predictor of participant attrition using Firth’s penalized likelihood logistic regression models. As displayed in Table 5, participants with a greater history of sexual abuse were more likely to drop out of the intervention. None of the other trauma variables significantly predicted participant attrition.

#### Qualitative reports

A few participants described MRAE during the MedEx-I that included references to trauma. One participant described a distressing experience during a guided focused attention meditation:

*“It was that horrible one with the fingers. It was using your hands as an anchor. […] That was very triggering for me. I have a history of sexual abuse, and during that one I had to stop it. Because…I…felt…very trapped. And that was really…distressing, so I stopped.” (Age 59, F)*

Another participant described intrusive traumatic memories arising during meditation:

*“I did find…uncomfortable feelings, memories, emotions coming up. The first time, it was very much a surprise…just sitting there and noting and hearing, and then all of a sudden something would pop into my head, and I’d get tearful and my palms might get sweaty.” (Age 58, F)*

Finally, a third participant shared that using noting practices during periods of anxiety amplified her anxious feelings, leading her to choose a different practice:

*"I think when I do experience anxiety and I try to note through it, it’s kind of like a positive feedback loop and it just…makes me more anxious. During those times, I try to focus my meditation on other things that are going on besides my body, [and] I try to [use hearing and seeing as anchors].”* (Age 19, F)

## Discussion

The present paper investigated the impact of childhood trauma history and subclinical PTSD symptoms on MBP treatment outcomes, side effects, and adverse effects across two clinical trials. Study 1 found that childhood trauma led to worse outcomes for both MBCT and control groups. Within this study, estimates of effect size for childhood trauma indicated that when participants had high levels of childhood trauma, the effect of childhood trauma on depression outcomes was greater than the effect of the treatment condition vs control group. Furthermore, trends in the data suggested that the MBCT group was more impacted by childhood trauma (physical, emotional, sexual abuse and total score) than the control group (sexual abuse and total score only).

Study 2 expanded on the results of study 1 by using a larger sample, a more established measure of childhood trauma, and investigating impacts on MRSEs and MRAEs in addition to depression outcomes. In this study, childhood trauma was associated with worse MBP depression outcomes when depression was assessed via self-report but not when depression was assessed by clinicians. Specifically, childhood sexual abuse, emotional neglect, and CTQ total score were associated with less improvement or worsening in self-reported depression symptoms throughout the intervention and follow-up period. This finding provides confirmatory evidence for Study 1 regarding total history of childhood trauma and sexual abuse while using a larger sample and validated measure of childhood trauma. Study 2 also found that childhood trauma and/or subclinical PTSD symptoms were predictive of MRSEs, MRAEs, LBEs, and participant dropout. Specifically, nearly all trauma variables included in this study were predictive of a greater occurrence of MRSEs and negative valence MRAEs, while total childhood trauma, childhood emotional abuse, sexual abuse, current subclinical PSTD, and past PTSD were predictive of negative impact MRAEs and/or LBEs. Lastly, history of childhood sexual abuse was associated with participant dropout. Taken together, results suggest that meditation practice may at times be destabilizing for those with a trauma history, leading to increased risk for adverse effects and/or participant attrition.

### Childhood trauma and MBCT depression outcomes

That individuals with active depression and a history of childhood trauma had worse MBP depression treatment outcomes is consistent with the pattern of previous findings. Such findings indicate that childhood trauma predicts *poorer* outcomes in MBCT treatment for active depression [13, 14] yet *better* outcomes when MBCT is used as a relapse prevention program in remitted individuals who are not currently depressed [11, 12]. This pattern is in line with the wider literature on the impact of childhood trauma on depression treatment, such that childhood trauma generally predicts worse depression outcomes in treatments for active depression [7]. It is not clear, however, if these worse outcomes are specific to the effects of mindfulness training or reflect a more general pattern. The present findings on the relationship between childhood trauma and MRAE suggests that this relationship is specific to mediation. However, in contrast, one study comparing MBCT to treatment as usual for actively depressed individuals found that childhood trauma was associated with worse depression symptoms at follow-up across both groups, but that this effect was lessened in the MBCT group compared to the treatment as usual group [44]. Therefore, further research is needed to clarify whether MBPs for active depression perform better or worse than other treatment approaches for individuals with childhood trauma.

Childhood sexual abuse was the only subscale that was associated with worse depression outcomes in both studies. In study 2, it was also associated with greater attrition and a greater occurrence of MRSE, negative valence MRAEs, and negative impact MRAEs, indicating that it had a direct negative interaction with meditation practice. Importantly, sexual trauma tends to be highly associated with the body, which is often the focus of meditative practices. Among survivors of childhood sexual trauma, body awareness has been found to trigger strongly aversive emotions such as anxiety, shame, guilt, disgust, and anger, as well as dissociative states [45, 46]. Indeed, the qualitative excerpts presented in the current study illustrate the extent to which a focus on body sensations may be distressing for individuals with a history of sexual trauma. Though exposure to distressing trauma-related emotions is a key component of many effective treatments for PTSD [47], individuals who encounter such emotions within meditation practice in a program designed to treat depression or stress may not be sufficiently prepared or supported to derive therapeutic benefit from them.

Interestingly, in study 2, the association between trauma history and worse depression outcomes was only found using the self-reported QIDS and not the clinician rated IDS. Clinician-rated depression scales have been found to produce larger effect sizes than self-reported depression scales [42], possibly because participants inflate their symptoms at baseline and minimize them at post-treatment in clinician administered interviews in order to show successful treatment, but are more honest in responding to self-report questionnaires [48]. Known as the “good subject effect,” this could have occurred as a result of demand characteristics, the “hype” surrounding meditation research, and social desirability bias [49, 50]. Indeed, in our sample, more participants exhibited an increase in depression symptoms as measured by the QIDS as opposed to the IDS after treatment and at follow-up.

### Childhood trauma and meditation-related adverse effects

Individuals with a trauma history endorsed more MRSEs and MRAEs in study 2. These findings are in line with established research on relationships between altered or dissociative states of consciousness and a history of childhood trauma and/or current trauma symptoms [27, 51–53]. Within this literature, PTSD has been conceptualized as fundamentally a disorder of affect arousal regulation [53], such that dysregulated arousal can lead to altered or dissociative states. Although participants endorsed a wide range of side effects and adverse effects within study 2, each of which may have their own specific mechanisms, trauma and meditation may interact through at least two main arousal-related pathways: hyperarousal (or traumatic reexperiencing) and hypoarousal (or dissociation).

#### Hyperarousal and traumatic re-experiencing

The most common MRSE and negative valence MRAE experiences, as reported in detail in Britton, Lindahl [18], were perceptual hypersensitivity, vivid imagery and thoughts, somatosensory changes, traumatic reexperiencing, anxiety and panic, affective lability, and agitation–all forms of nervous system hyperarousal [52]. Virtually all of the trauma variables in study 2 were associated with these variables, indicating that childhood trauma, past PTSD diagnoses, and current subclinical PTSD symptoms are associated with states of meditation-related hyperarousal and emotional distress. This replicates the findings of Zhu, Wekerle [28], who found that both trauma history and trauma-related symptoms predicted distressing experiences and traumatic reexperiencing as occurring during meditation practice. It is important to note that experiences categorized as MRSE were not necessarily distressing and experiences categorized as negative valence MRAE were not necessarily impairing. Although some hyperaroused states could result in negative impacts and/or be constitutive of LBEs (e.g., traumatic reexperiencing, anxiety and panic, insomnia, agitation), most instances did not result in prolonged impairment and some may have contributed to positive participant outcomes [18].

Meditation practice can lead to a sensitization of the nervous system and hyperaroused states through multiple possible pathways. According to Lindahl, Britton [54], these include: 1) sensitization through attentional training and 2) somato-affective amplification through body awareness. Through the first pathway, meditation is hypothesized to lead to increased arousal through its effects on sustained attention and the overlap between the neural processes underlying attention and arousal [54]. Given that baseline levels of arousal tend to already be elevated within traumatized individuals [55], a meditation-related increase in attention and arousal could especially result in hyperaroused states for individuals who have experienced significant trauma. In the second pathway, the body awareness component of meditation could lead to an amplification of affective states and emotional arousal that is mediated by body awareness and activation of the insular cortex [54, 56, 57]. The insula is related to body awareness and associated with both mindfulness training [58–60], and traumatic re-experiencing [61–63]. Since negative affective states are a characteristic of PTSD and are more common among individuals with a history of childhood trauma [64], a meditation-related amplification of such states could result in a relationship between trauma history and meditation-related hyperarousal.

The nature of traumatic memories and mindfulness practice may also interact in a related mechanism. According to dual representation theory [65], traumatic memories are characterized by an imbalance between sensory representations and contextual representations, such that sensory components (body sensations, emotions, images, smells) are increased at the expense of the memory’s contextualization and narrative. Similarly, mindfulness practice involves focusing on sensory data and instructions to “abandon conceptual judgments and narrative stories” [66]. As a result, mindfulness practice may amplify and exacerbate this trauma-related overemphasis on sensory representations rather than correcting it. An exclusive focus on sensory information runs contrary to prevailing models of therapy for PTSD, which emphasize the elaboration and integration of the trauma memory into contextual representations of time, place, meaning, and narrative [67]. Therefore, a trauma-informed modification to an MBP might involve the addition of a narrative component to the nonconceptual sensory awareness of mindfulness practice. For example, participants could be encouraged to speak or write about past memories or emotional experiences that arise during their meditation practice.

#### Hypoarousal and dissociation

As Britton, Lindahl [18] reports in detail, many of the experiences that were constitutive or predictive of LBEs, such as executive dysfunction, emotional blunting, self-disturbances, time-space distortions, and derealization, are common characteristics of hypoarousal or dissociation. Dissociation involves a detachment and compartmentalization of mental and emotional experiences that should otherwise be integrated [27]. Trauma-related dissociative states are less common than states involving hyperarousal and are believed to function as a way of escaping overwhelming levels of distress [27, 51]. Within study 2, total childhood trauma, childhood emotional abuse and current subclinical PTSD symptoms significantly predicted LBEs, indicating that these variables are associated with an increased risk of dissociative states as a result of meditation practice. These results are in line with Frewen, Brown [51], who found that childhood trauma led to an increased risk for trauma-related dissociation.

The hypothesized mechanisms for how meditation could lead to hypoarousal and dissociation have been described by Britton [57]. As with hyperarousal, two primary pathways have been proposed: 1) depersonalization through the cultivation of a detached self-perspective (e.g., decentering) within meditation practice and 2) emotional blunting through excessive prefrontal control of the limbic system within meditation practice. Though both of these well-documented processes are typically framed as leading to beneficial effects, overtraining may lead to dissociation in some people [57]. Individuals with a trauma history or symptoms of PTSD may already have experience entering into dissociative states, and thus may be particularly likely to do so via these mechanisms during meditation practice. A third but not mutually exclusive pathway leading from meditation to dissociation is that dissociation could occur as a defensive response to overwhelming emotional states that arise during meditation [27], such as those discussed previously in relation to somato-affective amplification and a meditation-related overemphasis on nonconceptual sensory awareness [54, 57]. Trauma-informed modifications to MBPs to prevent dissociation could include psychoeducation about the signs and symptoms of dissociation so that participants can learn to self-monitor and adjust their practice if dissociative symptoms arise.

### Limitations

Limitations for both studies include small sample sizes involving predominately female, white, and educated populations. As a result, these findings may not generalize to all individuals with a trauma history that participate in MBPs. An important line of future research would be to investigate whether similar results are found within underprivileged or marginalized populations, as such populations may experience a greater level of childhood trauma [68]. Additionally, individuals with a current diagnosis of PTSD were excluded from participating in study 2. Therefore, these results cannot be generalized to patients with current PTSD, despite the fact that current subclinical PTSD was included.

Limitations specific to study 1 include the use of a non-validated, exploratory measure of childhood trauma and data that was collected from 2004 to 2005. Given that the trauma variable was constructed post-hoc from semi-structured interview data, it is unlikely to have consistently captured the frequency and severity of participants’ childhood trauma experiences. However, this limitation was corrected by the inclusion of the validated CTQ in study 2 and the similarity in findings between the two studies. Additionally, the time that has lapsed since study 1 recruitment could impact its generalizability to contemporary settings, though study 2 was conducted more recently with a similar pattern of results.

A limitation within both studies is the lack of certainty about whether the effect of trauma was specific to the MBP. In study 1, interaction effects between trauma variables and condition (MBCT vs. control) were underpowered and either non-significant or not possible to calculate, while in study 2 there was no non-meditation or no-treatment control group. Therefore, results could reflect simply the passage of time, with or without treatment. While the presence of treatment-specific effects are suggested by the findings in study 1 that: 1) a greater number of trauma variables predicted worse depression scores in the treatment condition than in the control condition, as well as the findings in study 2 that: 2) trauma variables predicted MRAE, further studies should investigate this question using a larger sample and control group. What is clear from these data is that individuals with childhood trauma may have more difficulty with MBPs, and this knowledge may help teachers provide more targeted support.

### Implications for MBPs

These data have significant clinical implications. First, we recommend that MBPs for active depression include a history of childhood abuse or neglect and current PTSD symptoms in screening procedures. Unmodified MBP interventions targeted toward depression or stress may not be the most appropriate treatments for such individuals [50]; however, if they do participate in these programs, they should be identified and monitored closely during treatment. On the other hand, participants with active PTSD *were* screened out within both of the present studies but more subtle forms of trauma still predicted overall outcomes, MRAEs and attrition. This finding suggests that making all MBPs trauma-inclusive is likely to be more useful than screening out or creating separate trauma-sensitive programs for trauma survivors with PTSD, as prevalence rates for lifetime trauma exposure (e.g., 80.8%) are estimated to be high within the general population [69]. A number of trauma-sensitive modifications have been suggested: shorter durations of meditation practice, smaller group sizes, and one-on-one conversations with the mindfulness teacher about triggers and special requests, among others [70, 71]. Recent MBPs administered to trauma populations have successfully implemented such modifications. For example, in an adaptation of MBCT for adult-onset PTSD, meditation periods were no longer than 20 minutes and PTSD symptoms were discussed in class [72]. In an MBSR intervention for a nonclinical population of childhood sexual abuse survivors, language was adopted to emphasize an individual’s choice to participate in a meditation [16]. Finally, some researchers have suggested exclusion or modification of the body scan meditation especially for survivors of childhood sexual abuse [13, 72]. Trauma-informed MBCT interventions have been developed and should be compared with standard MBCT regarding outcomes and adverse effects.

## Conclusion

In summary, the present studies suggest that childhood trauma, especially childhood sexual abuse, may be associated with worse depression outcomes and increased attrition in MBPs for active depression. Childhood trauma and past and current PTSD symptoms may also be associated with MRSE and MRAE, such that childhood emotional abuse and current subclinical PTSD symptoms are likely to be most associated with LBEs. These findings provide support for the adoption of trauma-informed modifications to standard MBPs.

## Supporting information

S1 File Study 1 methods: Calculation of CTQ-BAS. (DOCX)

S2 File Study 2 methods: Missing data, attrition, and outliers. (DOCX)

S3 File Study 2 methods: More information about statistical models. (DOCX)

S4 File Study 2 results: Accounting for nested data structure and distributional assumptions. (DOCX)

S5 File Study 2 Results: Growth curve model construction. (DOCX)

S6 File Study 2 results: Controlling for the effects of trauma variables on depression intercept. (DOCX)

S7 File Study 1 data. (XLSX)

S8 File Study 2 data. (XLSX)

S1 Table Study 1 methods: Correspondence between CTQ items, coding categories, and their occurrence rates. (DOCX)

S2 Table Study 1 results: Trauma variables as predictors of pre-to-post BDI changes by condition. (DOCX)

S3 Table Study 1 results: Trauma variables as predictors of participant attrition. (DOCX)

S4 Table Study 2 results: Trauma variables as predictors of intervention-related changes in depression. (DOCX)
