# Supplementary material for: Childhood trauma and subclinical PTSD symptoms predict adverse effects and worse outcomes across two mindfulness-based programs for active depression
Source: PLoS One. 2025 Jan 30;20(1):e0318499. doi: 10.1371/journal.pone.0318499 (PMC11781677; doi:10.1371/journal.pone.0318499)
Supplement: S1 File — (DOCX) [file pone.0318499.s001.docx]

**S1 File**

Study 1 Methods: Calculation of CTQ-BAS

A directed qualitative analytic approach tailored for this study was used to extract an index of early adversity for subsequent analyses. To capture as much information as possible, adverse events identified during the SCID-IV interview, as well as responses to the self-report measure of potentially traumatic events from the personal history form, were examined for each participant and double coded by two members of the research team. Codes corresponding with types of adverse events were generated by examining established measures of early adverse experiences (Bernstein et al., 1994; Felitti et al., 1998; Gray et al., 2004; Taylor et al., 2004). Codes for the initial 20 participants were independently assigned by two coders, then discussed and reconciled as part of training. Among the remaining participants, coder agreement equaled 97.03% across all categories. To align with prior research examining early adversity as a predictor of response to MBCT (Williams et al., 2014), which used the Childhood Trauma Questionnaire (CTQ; (Bernstein et al., 1994), this list of codes was examined to identify categories that correspond with the CTQ (see S1 Table for list of codes). These codes were summed for a total Childhood Trauma Questionnaire-Based Adversity Score (CTQ-BAS) and four CTQ-BAS subscales (physical abuse, emotional abuse, sexual abuse, and physical neglect). These variables represented qualitatively coded proxies for the CTQ and its subscales and served as independent variables in subsequent analyses.

**References**

Bernstein, D. P., Fink, L., Handelsman, L., Foote, J., Lovejoy, M., Wenzel, K., Sapareto, E., & Ruggiero, J. (1994). Initial reliability and validity of a new retrospective measure of child abuse and neglect. *Am J Psychiatry*, *151*(8), 1132-1136. <https://doi.org/10.1176/ajp.151.8.1132>

Felitti, V. J., Anda, R. F., Nordenberg, D., Williamson, D. F., Spitz, A. M., Edwards, V., Koss, M. P., & Marks, J. S. (1998). Relationship of childhood abuse and household dysfunction to many of the leading causes of death in adults. The Adverse Childhood Experiences (ACE) Study. *Am J Prev Med*, *14*(4), 245-258. <https://doi.org/10.1016/s0749-3797(98)00017-8>

Gray, M. J., Litz, B. T., Hsu, J. L., & Lombardo, T. W. (2004). Psychometric properties of the life events checklist. *Assessment*, *11*(4), 330-341. <https://doi.org/10.1177/1073191104269954>

Taylor, S. E., Lerner, J. S., Sage, R. M., Lehman, B. J., & Seeman, T. E. (2004). Early environment, emotions, responses to stress, and health. *J Pers*, *72*(6), 1365-1393. <https://doi.org/10.1111/j.1467-6494.2004.00300.x>

Williams, J. M., Crane, C., Barnhofer, T., Brennan, K., Duggan, D. S., Fennell, M. J., Hackmann, A., Krusche, A., Muse, K., Von Rohr, I. R., Shah, D., Crane, R. S., Eames, C., Jones, M., Radford, S., Silverton, S., Sun, Y., Weatherley-Jones, E., Whitaker, C. J., . . . Russell, I. T. (2014). Mindfulness-based cognitive therapy for preventing relapse in recurrent depression: a randomized dismantling trial. *J Consult Clin Psychol*, *82*(2), 275-286. <https://doi.org/10.1037/a0035036>
