# Supplementary material for: Childhood trauma and subclinical PTSD symptoms predict adverse effects and worse outcomes across two mindfulness-based programs for active depression
Source: PLoS One. 2025 Jan 30;20(1):e0318499. doi: 10.1371/journal.pone.0318499 (PMC11781677; doi:10.1371/journal.pone.0318499)
Supplement: S2 File — (DOCX) [file pone.0318499.s002.docx]

**S2 File**

Study 2 Methods: Missing Data, Attrition, and Outliers

An intent to treat (ITT) approach was used for analysis that included all participants who were randomized to treatment, regardless of whether they dropped out after randomization. Eight participants dropped out of the study after randomization: one from FA, two from MBCT, and five from OM. Two participants, both in OM, dropped out before attending the first class due to moving away or no longer wanting to be part of the study. Two participants dropped out after class two (both MBCT) due to scheduling conflicts with work. Three participants dropped out after class three (all OM) due to the time commitment (*N* = 2) and increased stress due to the study (*N* = 1). Finally, one participant dropped out after class seven (FA) due to lacking “commitment to practice”. Missing data in longitudinal dependent variables were handled with the maximum likelihood procedure within mixed-effect models, while missing data in independent variables and dependent variables measured at a single time point were handled with listwise deletion.

All 104 participants had IDS and QIDS scores for at least one timepoint. However, one participant did not complete the CTQ and was thus excluded from the present analyses, resulting in a dataset of 103 participants. Data on meditation-related side-effects were only available for participants who completed the study (*N* = 96). The MRSE and Negative Valence MRAE variables contained no missing values, while the Negative Impact MRAE and LBE variables were present for 81 and 78 participants, respectively. Six participants skipped single items on the CTQ. CTQ summary scores were calculated as the mean of non-missing items, which was justified by the relative infrequency of missing responses (0.3% of all CTQ items). There were no missing SCID data.

Extreme outliers were identified in the physical abuse and sexual abuse CTQ subscales due to positively skewed and leptokurtic distributions. In order to reduce the impact of these outliers on statistical models, these two variables were winsorized by substituting values outside of the 95^th^ percentile range with values within that range. Sensitivity analyses indicated that windsorizing these variables had little effect on the study results.
