# Supplementary material for: Childhood trauma and subclinical PTSD symptoms predict adverse effects and worse outcomes across two mindfulness-based programs for active depression
Source: PLoS One. 2025 Jan 30;20(1):e0318499. doi: 10.1371/journal.pone.0318499 (PMC11781677; doi:10.1371/journal.pone.0318499)
Supplement: S3 File — (DOCX) [file pone.0318499.s003.docx]

**S3 File**

Study 2 Methods: More Information about Statistical Models

Longitudinal continuous outcomes were investigated using multilevel growth curve models in R using the nlme package and maximum likelihood estimation. As based on recommendations from Singer and Willett (2003), the time variable was measured in weeks such that post-course (8 weeks) was set to zero, causing the model intercept to equal the post-course mean. Before fixed predictors were added to these models, growth curve models were constructed using an exploratory approach to find the best fitting and most parsimonious model for the effects of time on each depression outcome. Linear and polynomial effects of time (as both fixed and random effects), as well as error and variance structures (autocorrelated error structure, compound symmetric error structure, exponent variance structure, etc.), were added to each model using deviance statistics to determine whether additional model parameters significantly improved fit. Parameters that did not significantly improve model fit were not retained within the models. Model residuals and fitted values were plotted to examine model assumptions. Interaction effects between effects of trauma and time were used to predict changes in depression. Interaction terms were added to models after the main effect of each trauma variable in order to control for the effect of trauma variables on the model intercept.

Count variable outcomes were investigated using multilevel generalized linear models in R using the lme4 package. As a precursor to model construction, the distribution of each dependent variable was investigated to distinguish whether a poisson or negative binomial distribution would provide the best fit to the data. Firth’s penalized likelihood logistic regression was used to predict binary outcomes. This approach was used because ordinary logistic regression is sensitive to low base rates and may produce biased estimates with low-probability data within a relatively small sample size. The logistf R package was used to run Firth’s penalized likelihood logistic regression models. These models did not account for nested data since existing software did not support this capability.

**References**

Singer, J. D., & Willett, J. B. (2003). *Applied longitudinal data analysis: Modeling change and event occurrence*. Oxford University Press. <https://doi.org/https://doi.org/10.1093/acprof:oso/9780195152968.001.0001>
