# Supplementary material for: Childhood trauma and subclinical PTSD symptoms predict adverse effects and worse outcomes across two mindfulness-based programs for active depression
Source: PLoS One. 2025 Jan 30;20(1):e0318499. doi: 10.1371/journal.pone.0318499 (PMC11781677; doi:10.1371/journal.pone.0318499)
Supplement: S4 File — (DOCX) [file pone.0318499.s004.docx]

**S4 File**

Study 2 Results: Accounting for Nested Data Structure and Distributional Assumptions

The first step in depression growth curve model construction was to investigate the structure of nested data for each depression outcome. Across all time points for QIDS depression scores, treatment type explained less than 0.01% of variance while group differences explained 1.94% of variance and participant differences explained 30.37% of variance. For IDS depression scores across all time points, neither treatment, group, or individual-level random intercepts explained more than 0.01% of variance. As a result, random intercepts at the level of the three treatment types were dropped from both models and the random group intercept was dropped from the IDS model.

Similarly, multilevel generalized linear models were fit to each count variable (MRSE, Negative Valence MRAE, and Negative Impact MRAE) to account for the nested data structure and distribution of the dependent variable. Likelihood ratio tests comparing models using a poisson distribution to models using a negative binomial distribution found that negative binomial distributions provided a better fit to the data for all three variables (*θ* MRSE = 2.05; *θ* Negative Valence MRAE = 1.51; *θ* Negative Impact MRAE = 0.76). Treatment type accounted for 1% of MRSE variance, <0.01% of Negative Valence MRAE variance, and 0.3% of Negative Impact MRAE variance. The nine groups accounted for 2% of MRSE variance, <0.01% of Negative Valence MRAE variance, and <0.01% of Negative Impact MRAE variance. As a result, both random effects were removed from the Negative Valence MRAE model and the group random effect was removed from the Negative Impact MRAE model.

In regard to the validity of CTQ scores, 17.48% had a mean score on the denial/minimization CTQ subscale of ≥ 3 and 4.54% had a mean denial/minimization score of ≥ 3.5.
