# Supplementary material for: Childhood trauma and subclinical PTSD symptoms predict adverse effects and worse outcomes across two mindfulness-based programs for active depression
Source: PLoS One. 2025 Jan 30;20(1):e0318499. doi: 10.1371/journal.pone.0318499 (PMC11781677; doi:10.1371/journal.pone.0318499)
Supplement: S5 File — (DOCX) [file pone.0318499.s005.docx]

**S5 File**

Study 2 Results: Growth Curve Model Construction

Following the steps outlined for depression models in S4 File, linear and polynomial effects of time were added to depression models as both fixed and random intercepts. Linear and quadratic time coefficients (fixed effects) significantly improved the fit of both models, indicating that both QIDS and IDS scores significantly changed across the time points measured (*ps* < .0001). Cubic time coefficients and random slope coefficients did not significantly improve the fit of either model (*ps* > .05) and thus were not retained. Finally, error and residual variance structures were tested for each model and retained if they significantly improved model fit. The IDS model was significantly improved by applying a power of the fitted value variance function (power = 0.70; *p* < .001). None of the error and residual variance structures significantly improved the QIDS model (*p* > .05). See the table below for the growth model parameters for each of these models before trauma predictors were added:

*Study 2: Depression* *growth curve model parameters without predictors of change*

|  | QIDS Depression | IDS Depression |
| --- | --- | --- |
| Variable | *B (SE)* | *B (SE)* |
| Intercept | 14.14*** *(0.36)* | 10.65*** *(0.59)* |
| Time | -0.22*** *(0.03)* | -0.95*** *(0.06)* |
| Time^2^ | 0.02*** *(<0.01)* | 0.08*** *(0.01)* |
| Variances |  |  |
| *σ²* residual | 7.19 | 0.68 |
| *σ²* participant intercept | 4.58 | 16.38 |
| *σ²* group intercept | 0.24 | - |
| Deviance | 1979.41 | 1902.15 |
| AIC | 1991.41 | 1914.15 |

*Note:* *p < .05, **p < .01, *** p < .001. The time variable was coded such that post-course scores represent the model intercept.
