# Supplementary material for: Childhood trauma and subclinical PTSD symptoms predict adverse effects and worse outcomes across two mindfulness-based programs for active depression
Source: PLoS One. 2025 Jan 30;20(1):e0318499. doi: 10.1371/journal.pone.0318499 (PMC11781677; doi:10.1371/journal.pone.0318499)
Supplement: S6 File — (DOCX) [file pone.0318499.s006.docx]

**S6 File**

Study 2 Results: Controlling for the Effects of Trauma Variables on Depression Intercept

In order to control for the effects of each trauma variable on depression levels across all time points (the model intercepts), trauma variables were first added to the depression growth models as predictors of the intercept (the main effect across all timepoints). All trauma variables significantly predicted greater levels of depression across all time points for both the QIDS and IDS measures of depression *(p* < .05)*:* CTQ total (QIDS: *χ*^2^ (1) = 13.73, p < .001; IDS: *χ*^2^ (1) = 10.59, p = .001), CTQ physical abuse (QIDS: *χ*^2^ (1) = 6.81, p = .009; IDS: *χ*^2^ (1) = 4.01, p = .045), CTQ emotional abuse (QIDS: *χ*^2^ (1) = 15.10, p < .001; IDS: *χ*^2^ (1) = 9.92, p = .002), sexual abuse (QIDS: *χ*^2^ (1) = 8.00, p = .005; IDS: *χ*^2^ (1) = 7.45, p = .006), CTQ physical neglect (QIDS: *χ*^2^ (1) = 10.00, p = .002; IDS: *χ*^2^ (1) = 8.70, p = .003), emotional neglect (QIDS: *χ*^2^(1) = 6.19, p = .013; IDS: *χ*^2^(1) = 5.23, p = .022), current subclinical PTSD (QIDS: *χ*^2^(1) = 12.66, p < .001; IDS: *χ*^2^(1) = 10.78, p = .001), and past PTSD diagnosis (QIDS: *χ*^2^(1) = 5.27, p = .022; IDS: *χ*^2^(1) = 14.02, p < .001).
