# Supplementary material for: Childhood trauma and subclinical PTSD symptoms predict adverse effects and worse outcomes across two mindfulness-based programs for active depression
Source: PLoS One. 2025 Jan 30;20(1):e0318499. doi: 10.1371/journal.pone.0318499 (PMC11781677; doi:10.1371/journal.pone.0318499)
Supplement: S1 Table — (DOCX) [file pone.0318499.s009.docx]

**S1 Table**

| Study 1 Methods: Correspondence between CTQ items, coding categories, and their occurrence rates | | | |
| --- | --- | --- | --- |
| CTQ Item | Coding Category | MBCT | Control |
| 1. I didn't have enough to eat. | Explicit mention of not enough to eat (under 18) | 0 | 0 |
| 2. I knew that there was someone to take care of me and protect me. | Mentions absence of person to care for and protect them (under 18) | 3 | 3 |
| 3. People in my family called me things like "stupid", "lazy", or "ugly" | Person was called names or derogated (under 18) | 0 | 0 |
| 4. My parents were too drunk or high to take care of the family. | Parent substance use interfered with care (under 18) | 0 | 0 |
| 5. There was someone in my family who helped me feel important or special.* | Person felt important or special (under 18) | 0 | 0 |
| 6. I had to wear dirty clothes. | Explicit mention of dirty clothes (under 18) | 0 | 0 |
| 7. I felt loved.* | Person felt loved (under 18) | 0 | 0 |
| 8. I thought that my parents wished I had never been born.* | Thought parents did not want them (under 18) | 0 | 0 |
| 9. I got hit so hard by someone in my family that I had to see a doctor or go to the hospital. | Required medical attention for domestic violence (under 18) | 0 | 0 |
| 10. There was nothing I wanted to change about my family** | n/a |  |  |
| 11. People in my family hit me so hard that it left me with bruises or marks. | Physical abuse left bruises or marks (under 18) | 0 | 0 |
| 12. I was punished with a belt, a board, a cord (or some other hard object). | Punishment with object (under 18) | 0 | 0 |
| 13. People in my family looked out for each other.* | Positive statement that people in family took care of/supported each other (under 18) | 0 | 0 |
| 14. People in my family said hurtful or insulting things to me. | Hurtful things were said (not names/put-downs) (under 18) | 0 | 0 |
| 15. I believe that I was physically abused. | Endorsed "physical abuse" (under 18) | 4 | 2 |
| 16. I had the perfect childhood.** | n/a | 0 |  |
| 17. I got hit or beaten so badly that It was noticed by someone like a teacher, neighbor, or doctor. | Physical abuse noticed by someone in environment (under 18) | 0 | 0 |
| 18. Someone in my family hated me. | Someone in family hated the participant (under 18) | 0 | 0 |
| 19. People in my family felt close to each other.* | Positive statement that family members felt close to each other (under 18) | 0 | 0 |
| 20. Someone tried to touch me in a sexual way or tried to make me touch them. | Attempted sexual violation (under 18) | 9 | 3 |
| 21. Someone threatened to hurt me or tell lies about me unless I did something sexual with them. | Sexual manipulation or coercion (e.g. blackmail, threats) (under 18) | 0 | 0 |
| 22. I had the best family in the world.** | n/a |  |  |
| 23. Someone tried to make me do sexual things or watch sexual things. | Attempt to make person do or watch sexual things (under 18) | 1 | 0 |
| 24. Someone molested me (took advantage of me sexually). | Endorsed molestation or having been taken sexual advantage of (under 18) | 8 | 3 |
| 25. I believe that I was emotionally abused. | Endorsed "emotional abuse" (under 18) | 5 | 5 |
| 26. There was someone to take me to the doctor if I needed it.* | Mentions person who took them to appointments/tasks (under 18) | 0 | 0 |
| 27. I believe that I was sexually abused. | Endorsement of "sexual abuse" (under 18) | 8 | 5 |
| 28. My family was a source of strength and support.* | Positive statement that family was source of strength or support (under 18) | 0 | 0 |

*Note*. Items with ** are intended to detect response bias in the original CTQ questionnaire – they have been included here for completeness but were not applied in our coding systems because of unlikelihood they would be volunteered during the interview. Items with * are reverse-scored in the original CTQ questionnaire. However, as they contain positive statements about supportive experiences growing up and the SCID-IV interview and notes focused on adverse experiences, these were recorded but not used in the final analyses. Sensitivity analyses comparing this analysis with an analysis of sum scores that included these reverse-scored items did not meaningfully change the results on any parameters.
