# Supplementary material for: Childhood trauma and subclinical PTSD symptoms predict adverse effects and worse outcomes across two mindfulness-based programs for active depression
Source: PLoS One. 2025 Jan 30;20(1):e0318499. doi: 10.1371/journal.pone.0318499 (PMC11781677; doi:10.1371/journal.pone.0318499)
Supplement: S2 Table — (DOCX) [file pone.0318499.s010.docx]

**S2 Table**

Study 1 Results: Trauma Variables as Predictors of Pre-to-Post BDI Changes by Condition

|  | ∆BDI in MBCT Condition | | | | ∆BDI in Control Condition | | | |
| --- | --- | --- | --- | --- | --- | --- | --- | --- |
| Predictors: | *R*^2^ | *b* | 95% CI | | *R*^2^ | *b* | 95% CI | |
|  |  |  | *LL* | *UL* |  |  | *LL* | *UL* |
| CTQ-BAS Total | **.37** | **2.79**** | **1.30** | **4.11** | **.25** | **2.00*** | **-0.50** | **3.49** |
| CTQ-BAS Physical Abuse | **.27** | **11.09**** | **4.10** | **18.43** | <.01 | -1.61 | -4.47 | 1.06 |
| CTQ-BAS Emotional Abuse | **.17** | **8.83*** | **2.36** | **18.35** | 0.01 | 1.23 | -4.24 | 8.20 |
| CTQ-BAS Sexual Abuse | **.21** | **2.41*** | **0.42** | **4.40** | **.39** | **3.32**** | **-1.04** | **4.31** |
| CTQ-BAS Physical Neglect | .01 | 3.62 | 0.91 | 6.40 | <.01 | 0.96 | -7.38 | 9.43 |

Note: *N* = 26 for MBCT condition analyses and 19 for control condition analyses. Each predictor was entered into a single regression model to predict ∆BDI. CTQ-BAS = Childhood Trauma Questionnaire-Based Adversity Score; CI = confidence interval; LL = lower limit; UL = upper limit; Coefficients were calculated through bootstrapping with 5,000 iterations.

*p < .05, **p < .01, *** p < .001.
