# Supplementary material for: Childhood trauma and subclinical PTSD symptoms predict adverse effects and worse outcomes across two mindfulness-based programs for active depression
Source: PLoS One. 2025 Jan 30;20(1):e0318499. doi: 10.1371/journal.pone.0318499 (PMC11781677; doi:10.1371/journal.pone.0318499)
Supplement: S3 Table — (DOCX) [file pone.0318499.s011.docx]

**S3 Table**

Study 1 Results: Trauma Variables as Predictors of Participant Attrition

|  | Attrition | | |
| --- | --- | --- | --- |
| Predictors: | *OR* | 95 % CI | |
|  |  | *LL* | *UL* |
| CTQ-BAS Total | 1.32 | 0.86 | 2.02 |
| CTQ-BAS Physical Abuse | 4.19 | 0.61 | 24.81 |
| CTQ-BAS Emotional Abuse | 3.99 | 0.76 | 20.29 |
| CTQ-BAS Sexual Abuse | 1.15 | 0.61 | 1.98 |
| CTQ-BAS Physical Neglect | 4.19 | 0.61 | 24.81 |

Note: *N* = 52 for all models. CTQ-BAS = Childhood Trauma Questionnaire-Based Adversity Score; CI = confidence interval; LL = lower limit; UL = upper; OR = Odds Ratio. All models used Firth’s Penalized Likelihood Logistic Regression with dichotomous dependent variables.

*p < .05, **p < .01, *** p < .001.
