# Supplementary material for: Childhood trauma and subclinical PTSD symptoms predict adverse effects and worse outcomes across two mindfulness-based programs for active depression
Source: PLoS One. 2025 Jan 30;20(1):e0318499. doi: 10.1371/journal.pone.0318499 (PMC11781677; doi:10.1371/journal.pone.0318499)
Supplement: S4 Table — (DOCX) [file pone.0318499.s012.docx]

**S4 Table**

Study 2 Results: Trauma Variables as Predictors of Intervention-Related Changes in Depression

|  | | QIDS Depression | | | | IDS Depression | | | |
| --- | --- | --- | --- | --- | --- | --- | --- | --- | --- |
| Predictors: | | *χ*^2^(2) | *b* | 95% CI | | *χ*^2^(2) | *b* | 95% CI | |
|  | |  |  | *LL* | *UL* |  |  | *LL* | *UL* |
| CTQ Total | | 5.11 |  |  |  | 0.09 |  |  |  |
|  | X time |  | **0.72*** | **0.09** | **1.35** |  | -0.35 | -1.90 | 1.20 |
|  | X time^2^ |  | -0.77 | -1.67 | 0.13 |  | -0.54 | -2.36 | 1.28 |
| CTQ Physical Abuse | | 2.15 |  |  |  | 0.22 |  |  |  |
|  | X time |  | 0.58 | -0.20 | 1.36 |  | -0.17 | -2.09 | 1.75 |
|  | X time^2^ |  | -0.50 | -1.62 | 0.62 |  | -0.97 | -3.24 | 1.30 |
| CTQ Emotional Abuse | | 2.41 |  |  |  | 0.31 |  |  |  |
|  | X time |  | 0.29 | -0.08 | 0.66 |  | -0.32 | -1.26 | 0.62 |
|  | X time^2^ |  | -0.23 | -0.76 | 0.30 |  | -0.02 | -1.10 | 1.06 |
| CTQ Sexual Abuse | | 5.14 |  |  |  | 0.03 |  |  |  |
|  | X time |  | **0.79*** | **0.03** | **1.55** |  | -0.17 | -2.07 | 1.73 |
|  | X time^2^ |  | **-1.15*** | **-2.25** | **-0.05** |  | 0.26 | -2.09 | 2.61 |
| CTQ Physical Neglect | | 1.36 |  |  |  | 1.14 |  |  |  |
|  | X time |  | 0.46 | -0.30 | 1.22 |  | -0.15 | -2.05 | 1.75 |
|  | X time^2^ |  | -0.46 | -1.58 | 0.66 |  | -0.30 | -2.57 | 1.97 |
| CTQ Emotional Neglect | | 6.26* |  |  |  | 0.39 |  |  |  |
|  | X time |  | **0.45*** | **0.10** | **0.80** |  | -0.11 | -0.97 | 0.75 |
|  | X time^2^ |  | **-0.51*** | **-1.02** | **0.00** |  | -0.39 | -1.39 | 0.61 |
| Current Subclinical PTSD | | 1.38 |  |  |  | 0.90 |  |  |  |
|  | X time |  | -0.31 | -1.39 | 0.77 |  | 0.71 | -2.09 | 3.51 |
|  | X time^2^ |  | -0.28 | -1.87 | 1.31 |  | -1.25 | -4.82 | 2.32 |
| Past PTSD | | 2.31 |  |  |  | 0.09 |  |  |  |
|  | X time |  | 0.71 | -0.21 | 1.63 |  | -0.69 | -3.02 | 1.64 |
|  | X time^2^ |  | -0.78 | -2.09 | 0.53 |  | -0.11 | -2.93 | 2.71 |

Note: *N* = 103 for all models. Interactions with effects of time refer to predictions of change. X time = interactions with the linear time coefficient; X time^2^ = interactions with the quadratic time coefficient; CI = confidence interval; LL = lower limit; UL = upper limit; CTQ = Childhood Trauma Questionnaire; PTSD = Post traumatic stress disorder. Time variables were divided by their standard deviation so that interactions with each exponent of time would be on the same scale. Chi square statistics refer to likelihood ratio tests for the comparison of each model with the same model without the interaction terms (trauma variables predicting intercept only).

*p < .05, **p < .01, *** p < .001.
